# Collaborative modeling key to improving outbreak response

**DOI:** 10.1073/pnas.2200703119

**Published:** 2022-03-23

**Authors:** Nicholas G. Reich, Evan L. Ray

**Affiliations:** ^a^School of Public Health and Health Sciences, University of Massachusetts Amherst, Amherst, MA 01003

During the COVID-19 pandemic, modeling and forecasting have informed public health response at the local, state, and national levels by improving situational awareness, providing estimates of key virus characteristics, and optimizing mitigation strategies (1).

While forecasting efforts often have been the most visible modeling outputs to the general public, as predictions are often highlighted by the media, other modeling has played an important role in the pandemic as well. In PNAS, Fox et al. detail an important and influential collaborative modeling effort that has supported real-time public health decision-making in Austin, TX, during the COVID-19 pandemic (2). The effort described by Fox et al. is notable both for its careful and accurate modeling as well as the in-depth collaboration, clearly built on a relationship of trust, with Austin city officials.

While this effort is exemplary and hopefully will serve as a model for future similar collaborative work, the paper also raises important questions about how this kind of effort can be scaled. Ideally many municipalities, including ones that are not fortunate enough to have a terrific academic modeling group in or near their city, could take advantage of the insights that models have to offer. Can state and national public health agencies support scalable modeling efforts so that every local and state government can take advantage of a wide range of insights from robust modeling efforts? Furthermore, in doing so, can we reduce the dependency of such an undertaking on one single modeling group, by relying on the successful use of collaborative modeling “hubs” that have sprouted up before and during the pandemic (3–10) and/or by supporting the development of modeling capacity within public health agencies?

## Interpreting the Results of This Work

There are many clear collaborative modeling successes described by Fox et al. (2). They describe the judicious use of real-time modeling whose outputs were tailored to specific needs based on conversations with city officials. From the outset, the group focused on metrics of the hospital system (new admissions, all hospital beds used, and intensive care unit [ICU] beds used), all key indicators of healthcare system stress. Importantly, they show very clear evidence that data on hospital admissions are strongly correlated with hospital and ICU bed use in the near future (on the scale of a week or two; see figure 1B of ref. 2). Not surprisingly, and likely due to changing trends in case reporting and care-seeking, case data showed substantially lower correlation and therefore were seen as a less useful “leading indicator” of future hospitalizations. As the authors summarize it, “COVID-19 hospital admissions provide a more accurate and timely indication of recent transmission and imminent healthcare usage.”

Another notable strength of this paper is the authors’ careful and honest evaluation of their model predictions. Fox et al. (2) appropriately claim success in places where their model provided accurate predictions but also point out places where their model showed less accuracy than desired. One important external validation step the authors take is to compare their model-estimated results on the cumulative fraction infected to an independent data source, the estimates of infection rates from serological studies published by the Centers for Disease Control and Prevention (CDC). While this is not a perfect ground-truth data source, as the CDC estimates have quite a lot of uncertainty and may have their own biases, it does provide an external “sanity check” on the model.

The modeling was not limited solely to predictive modeling, either. By estimating the reproduction number (a measure of the rate at which the pathogen is being transmitted) in real time, the University of Texas at Austin (UT) model provided immediate feedback to policy makers about the ways in which policies may have shifted the course of the pandemic in Austin. Additionally, having a group of experts in data processing and modeling who are monitoring the noisy pandemic data enables governments to operate with more confidence in the face of unforeseen disruptions or data anomalies, such as the unusual winter freeze event in Texas in early 2021, which came just after a large backlog of cases had been reported (figure 2F of ref. 2). These kinds of anomalies are commonplace in many locations, and having a dedicated group who is working to monitor and adjust data to account for such anomalies can be critically important to interpreting data appropriately in those situations.

Overall, the fact that these models “informed numerous time-sensitive policy decisions and response actions, including resource planning by local hospitals, urgent requests to state and federal agencies for additional surge resources, the launch and dismantling of alternative care sites to provide additional healthcare capacity, and numerous changes in the Austin-area COVID-19 alert stage to communicate and manage rising and declining risks” (2) shows the importance this effort played in allowing Austin to incorporate the strongest evidence possible into their decision-making.

## There Is Still a Lot to Learn

Looking forward, understanding which data sources are important and help improve real-time model accuracy is one of the most important lines of inquiry for epidemic modeling. The authors show results from a careful analysis demonstrating how incorporating mobility data improved the accuracy of their model. These results are useful but should also not be overinterpreted. The authors state that “our mobility-driven mechanistic model provides the best combination of accuracy and precision surrounding pandemic surges” in part because “removing the mobility covariate from our model significantly increases forecasting uncertainty” (2). However, further studies are needed to verify whether this result holds in other contexts. For example, it is possible that other modeling frameworks might account for uncertainty better than the one used in this paper, meaning that the addition of mobility data might not add substantial value. Additionally, the extent to which mobility data are a useful indicator for prediction may vary depending on the context. For example, in locations where vaccination uptake is high (this study was conducted largely prior to widespread vaccination) or where rates of mask wearing are also high, mobility may have a reduced role compared to what was seen here. Indeed, attenuated associations between mobility and COVID-19 transmission have been reported in the literature (11, 12). Although some have started to look into the question of what new data streams may be informative (13), more research is needed to establish what the most valuable real-time data streams are.

Another area where Fox et al. (2) introduce valuable ideas but ultimately leave us with open questions is surrounding the causal effects of different policies on COVID-19 transmission and subsequent trends in case, hospitalization, and death rates. The authors appropriately note that their “retrospective analysis of the Austin experience provides anecdotes regarding the impact of COVID-19 policies on risks” and are careful to not make statements that could be interpreted causally regarding the impact that specific policies or other dynamics had on COVID-19 transmission. Two years into the COVID-19 pandemic, this area of study remains underexamined, with limited numbers of studies doing formal causal inference analysis on the impacts of different interventions or exposures on COVID-19 transmission (14).

Relatedly, our ability to explain, even with the benefit of hindsight, why the case and hospitalization curves have followed the specific paths they did remains frustratingly limited. While new rises in cases have, in the last year, been somewhat predictable based on new variants emerging, there have not always been clear explanations for why rates have declined rapidly at important moments of peak transmission (for example, the quick downward trends in January 2021 or January 2022 in many locations in the United States).

## Making Models Work for Everyone

As robust modeling efforts like these that are deeply engaged with local governments become more commonplace, we must learn how to replicate the parts of the efforts that can easily be scaled, so that more municipalities and jurisdictions can reap the benefits of these modeling efforts. Especially as governments and officials are initially learning how to incorporate modeling into their work, it can be difficult to replace the “human” element in the relationship, where trust is established over time.

One route to making aspects of what the team at UT accomplished more broadly available is through a “modeling hub” approach that coordinates and aggregates model outputs from many modeling teams and produces results for many locations at once. Two modeling hubs that have supported decision-makers in the United States—the US COVID-19 Forecast Hub and the Scenario Modeling Hub—have provided, since April 2020 and December 2020, respectively, regular modeling updates that synthesize results from multiple groups (4, 5). (We, the authors of this piece, direct the US COVID-19 Forecast Hub, and the UT team has contributed forecasts to the Forecast Hub.) While these results, because they are generated for multiple locations at once, cannot be as individually tailored to the particular needs of a given jurisdiction as the ones from the UT effort, just the fact that such a resource exists is a testament to the vision of US CDC staff scientists who have worked for nearly a decade to build similar systems for annual influenza epidemics (15).

However, state and local public health agencies will continue to face questions for which modeling may provide valuable insights yet are not directly addressed by centralized modeling efforts. It will be valuable to build more modeling capacity within these agencies so that they can address such questions without necessarily relying on external modeling groups. Here again, national organizations such as the CDC and the Council of State and Territorial Epidemiologists could have a role to play in coordinating discussions to share best practices around the development and use of forecasts.

## A New Hope

In conclusion, the paper by Fox et al. (2) sets a very high bar in having conducted meaningful, collaborative science in real-time during a pandemic crisis. Making sure that the fruits of efforts like these can be made available to any local jurisdiction that wants them will require continued investment in data modernization, modeling technologies, and workforce development. Just as, if not more importantly, it will require collaboration between modeling groups (both in academia and industry) and local governments to ensure that the outcomes are meaningful and can be readily incorporated into decisions. These relationships must be cultivated in “peace time” and not only in the midst of the next epidemic or pandemic crisis. Both the US CDC, which has supported collaborative modeling hubs and in 2021 established a new Center for Forecasting and Analytics alongside its data modernization initiative, and the European Centre for Disease Prevention and Control, which has also supported modeling hubs over the last 2 y, are investing in efforts that can help make this a reality. Ultimately, success in this realm will combine the lessons learned from efforts like those described by Fox et al. (2) with the scalable technological frameworks established by modeling hubs. In this way, the integration of robust modeling outputs into local decision-making will hopefully become the norm rather than the exception.
